# Supplementary material for: Evaluating the performance of Plasmodium falciparum genetic metrics for inferring National Malaria Control Programme reported incidence in Senegal
Source: Malar J. 2024 Mar 5;23:68. doi: 10.1186/s12936-024-04897-z (PMC10916253; doi:10.1186/s12936-024-04897-z)
Supplement: Supplementary file 2 — Additional file 2. Additional tables and figures [file 12936_2024_4897_MOESM2_ESM.docx]

**
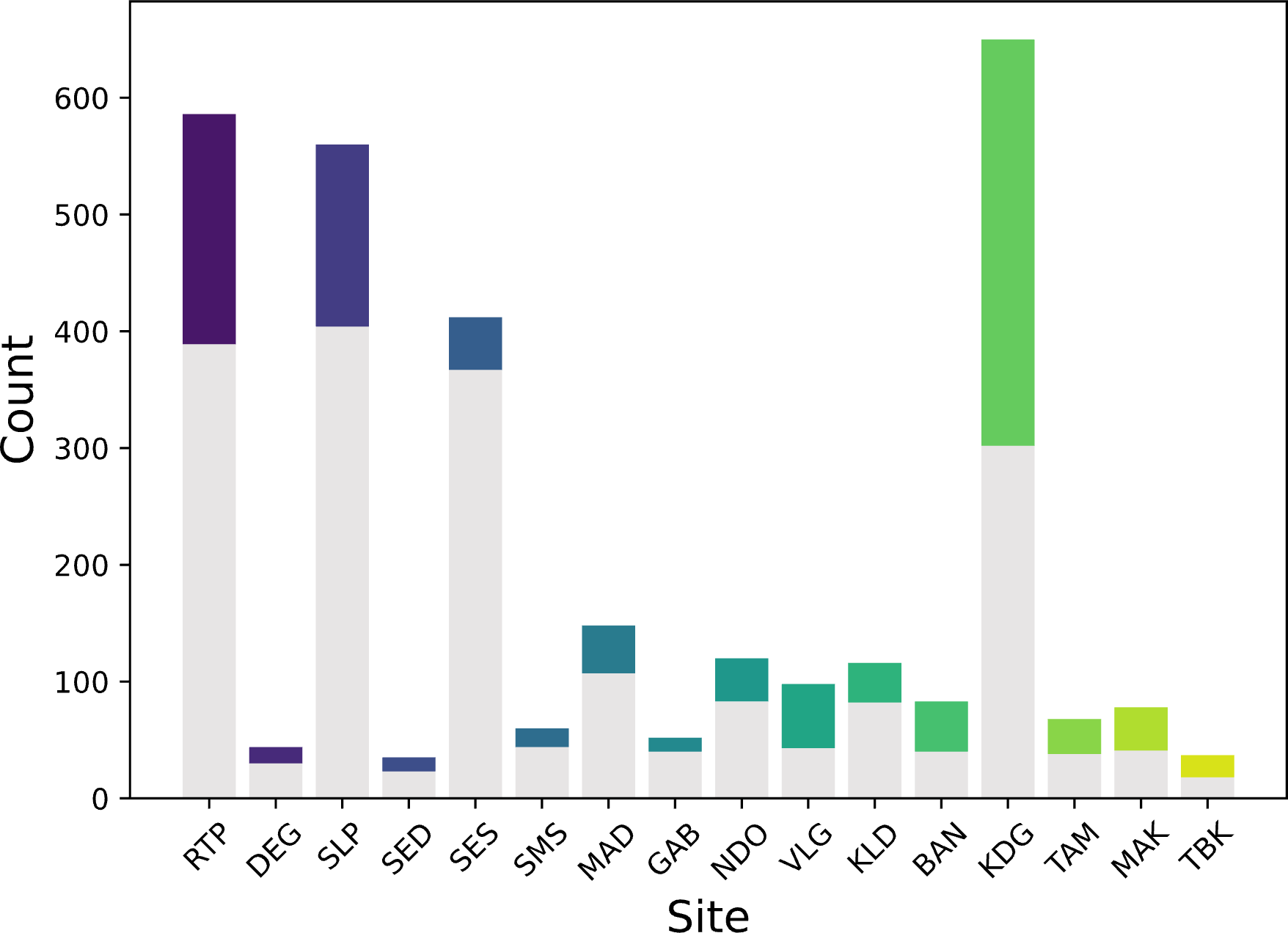
**

**Additional Fig. 1** The total number of genotyped samples collected at each site. Grey bars indicate monogenomic samples while coloured bars indicate polygenomic infections. The colour scheme used in this plot corresponds to the incidences in **Fig. 1A**.


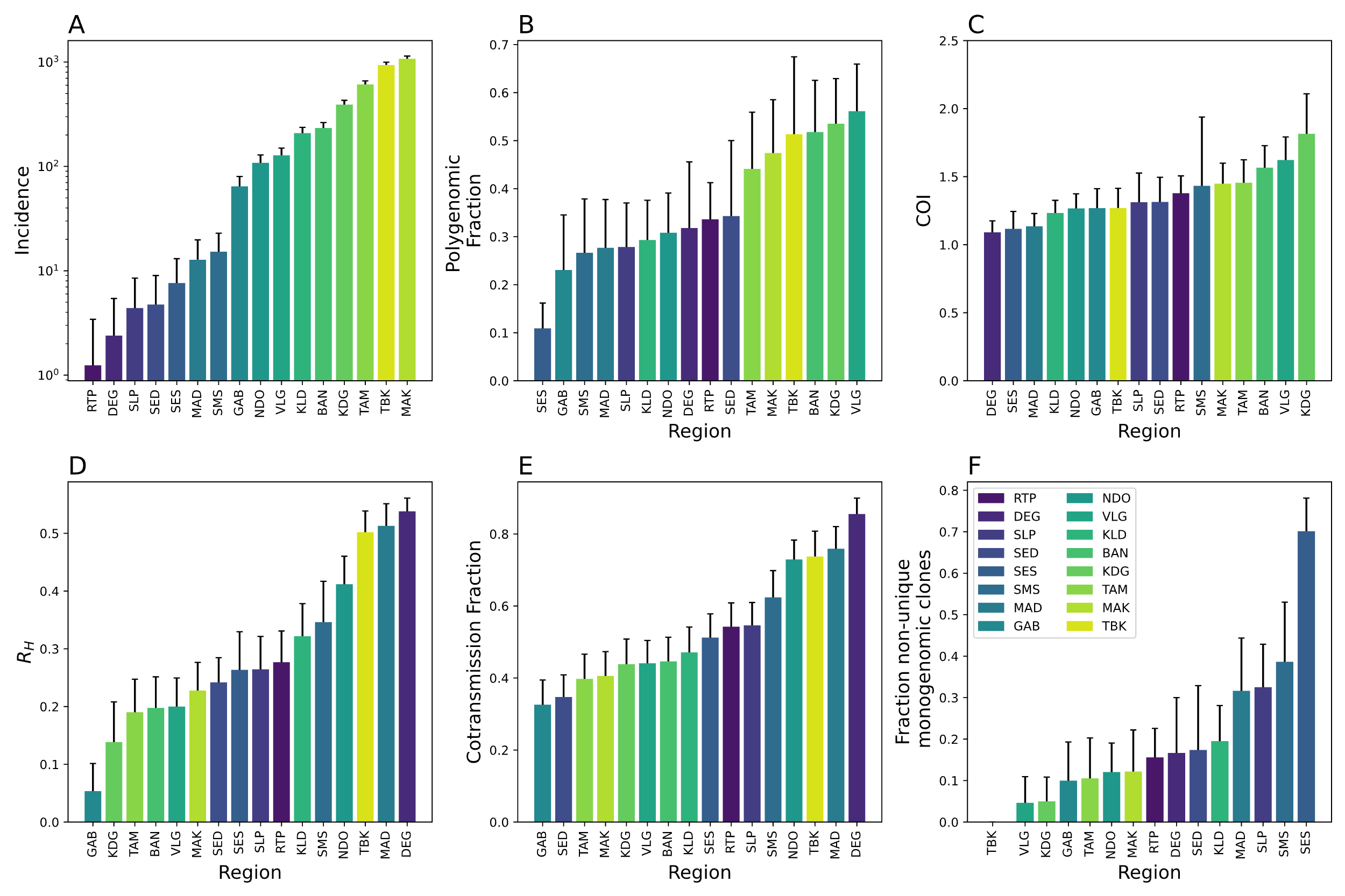


**Additional Fig. 2** Histograms of the genetic metrics used in this study, averaged across the sample years for each region. Error bars represent two standard deviations from the mean.


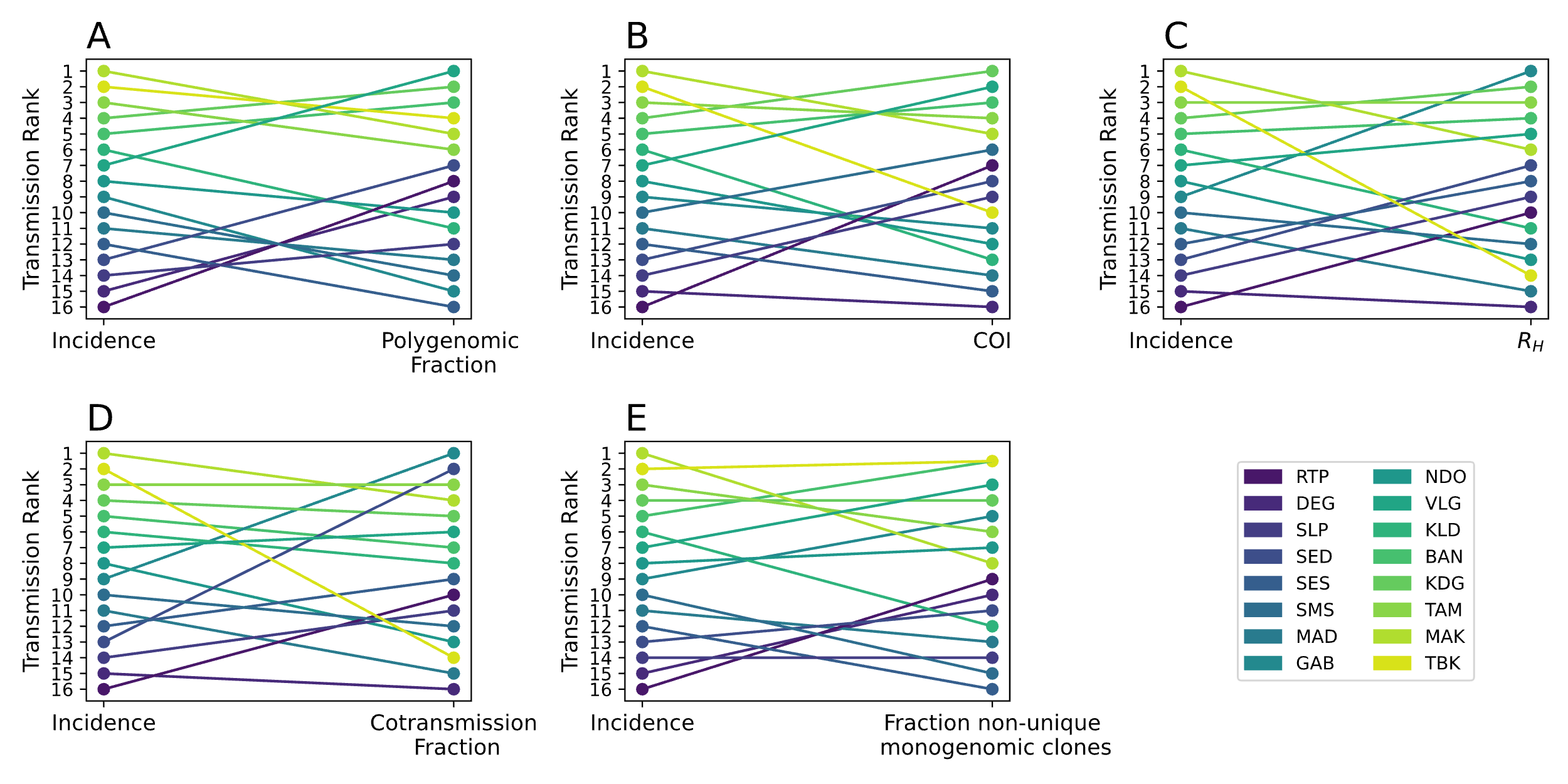


**Additional Fig. 3** Transmission rank switching for each of the five genetic epidemiology metrics examined in this study.


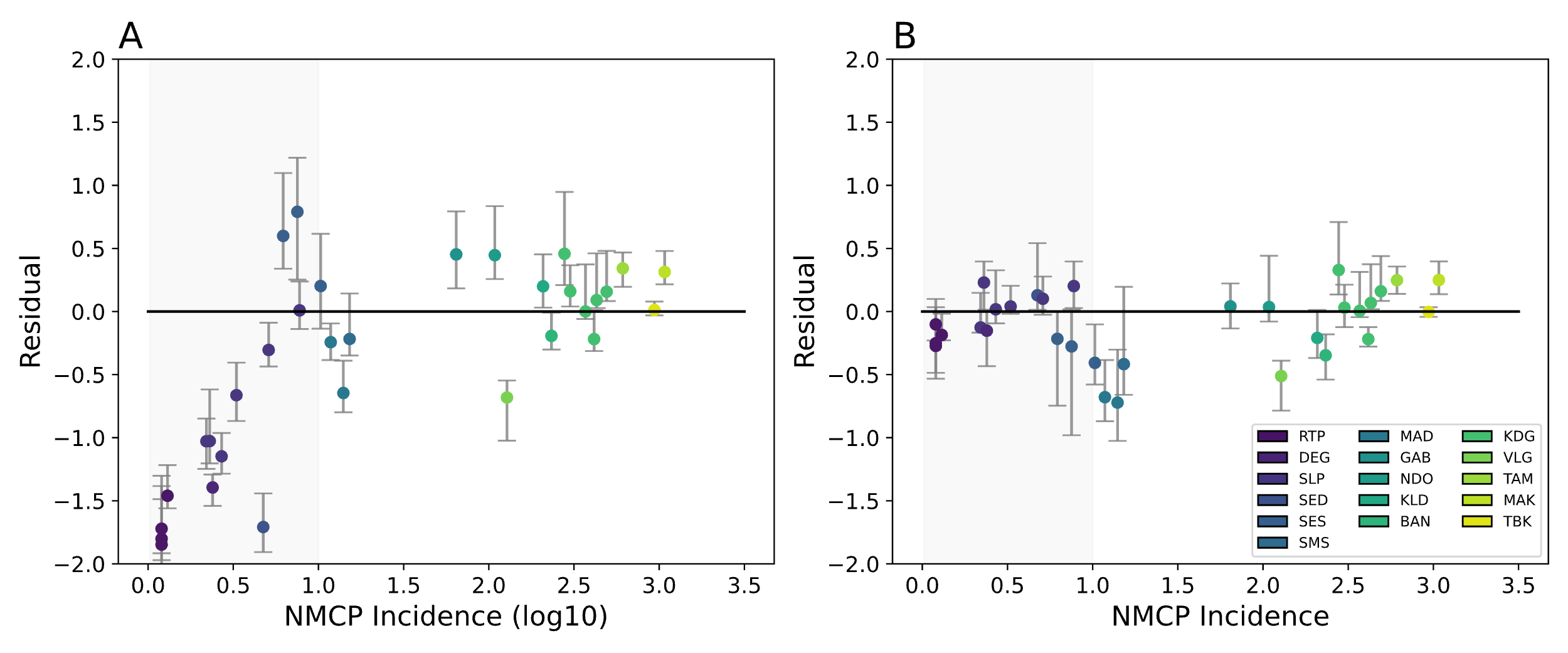


**Additional Fig. 4** Residual plot for the (**A**) GLM constructed using all five genetic metrics and (**B**) piecewise GLM that consists of GLM_below10_ and GLM_above10_. Residuals were calculated as the difference between the log10 NMCP-reported incidence and the log10 predicted incidence. Error bars show the 95% confidence interval generated through leave-one-out cross-validation. Positive values indicate the predicted incidence was lower than the reported incidence. Negative values indicate the predicted incidence was higher than the reported incidence. The grey shading highlights the data where the reported incidence was < 10‰. The legend applies to both **A** and **B**.


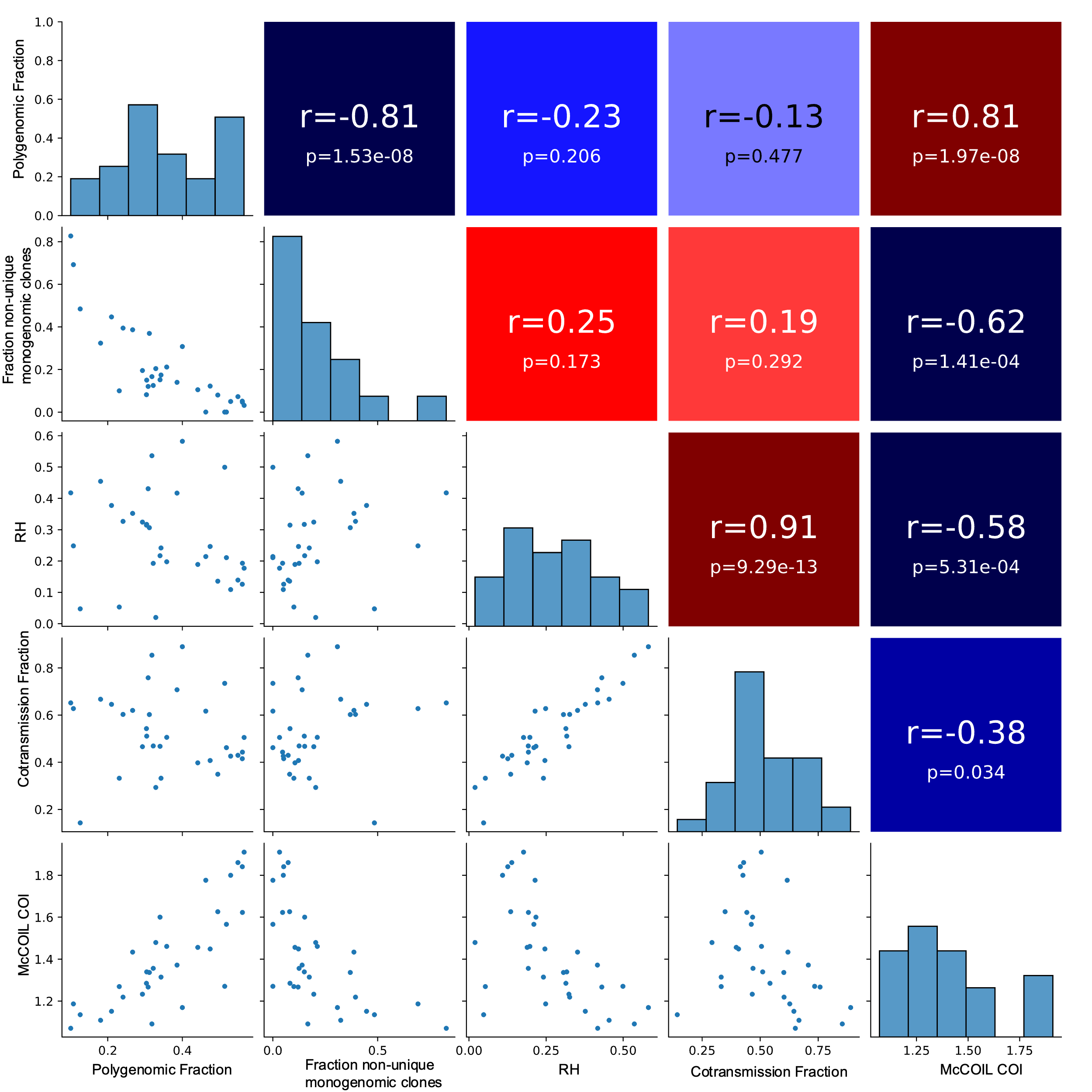


**Additional Fig. 5** Genetic metric covariance matrix. The upper triangle reports the Pearsons correlation coefficients and the associated *p*-values. The diagonal shows the distributions of each genetic metric and the lower triangle show various pairwise comparisons.


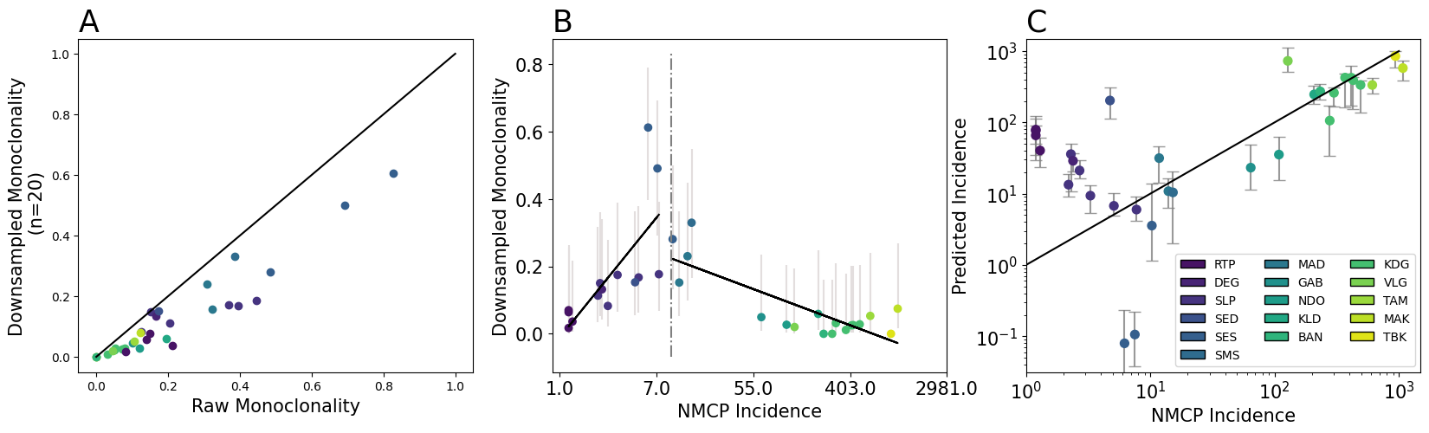


**Additional Fig. 6.** Effect of down-sampling the fraction of non-unique monogenomic clones. The fraction of non-unique monogenomic clones was re-estimated by randomly down-sampling the monogenomic infections to the smallest monogenomic infection count collected across all site-years. **A**) The fraction of non-unique monogenomic clones estimate using all the data (Raw) vs the down-sampled estimate. **B**) The down-sampled fraction of non-unique monogenomic clones against NMCP incidence. **C**) Model predictions using the down-sampled fraction of non-unique monogenomic clone estimates.


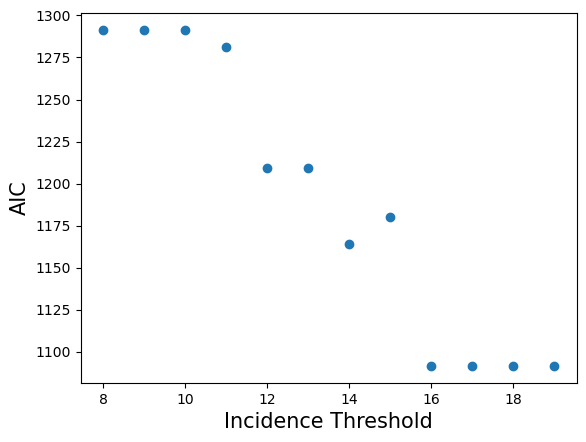


**Additional Fig. 7** Incidence threshold sweeps used in the piecewise GLM model. Lower AIC indicates better model fit. AIC was calculated from the average, leave-one-out cross validated log-likelihood.


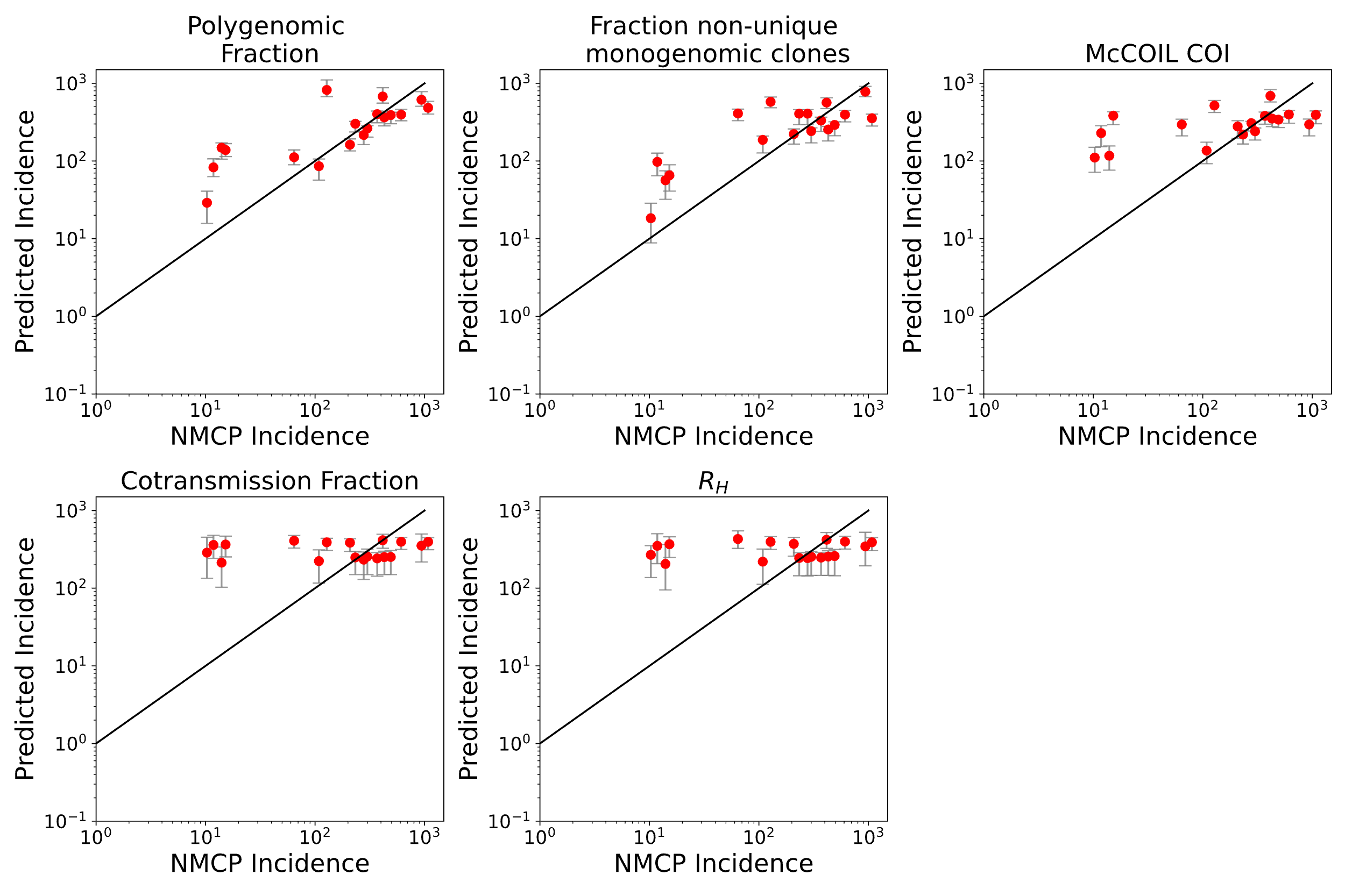


**Additional Fig. 8** Individual genetic metric model predictions using the GLM trained on data with incidence > 10‰. Error bars indicate the 95% confidence interval generated using leave-one-out cross-validation.


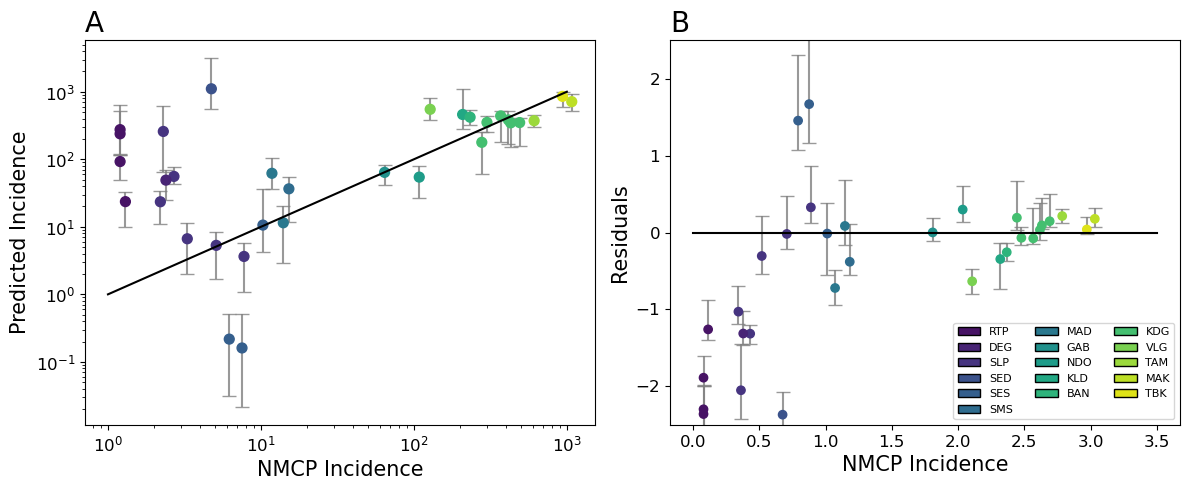


**Additional Fig. 9** Incidence predictions using the GLM trained on data with incidence > 10 ‰ (**A**) and their residuals (**B**). Error bars indicate the 95% confidence interval generated using leave-one-out cross-validation. For **B**, positive values indicate the predicted incidence was lower than the reported incidence. Negative values indicate the predicted incidence was higher than the reported incidence. The legend applies to both **A** and **B**.

**Additional Table 1** Number of successfully genotyped samples for each site, split by year.

| **Sample Site** | **Year** | **Successfully Genotyped Samples** |
| --- | --- | --- |
| RTP | 2012 | 70 |
| RTP | 2013 | 158 |
| RTP | 2014 | 115 |
| RTP | 2015 | 243 |
| DEG | 2019 | 44 |
| SLP | 2015 | 119 |
| SLP | 2016 | 137 |
| SLP | 2017 | 122 |
| SLP | 2018 | 73 |
| SLP | 2019 | 59 |
| SLP | 2020 | 50 |
| SED | 2019 | 35 |
| SES | 2018 | 111 |
| SES | 2019 | 102 |
| SES | 2020 | 199 |
| SMS | 2019 | 60 |
| MAD | 2019 | 83 |
| MAD | 2020 | 65 |
| GAB | 2019 | 52 |
| NDO | 2020 | 120 |
| VLG | 2019 | 98 |
| KLD | 2019 | 116 |
| BAN | 2020 | 83 |
| KDG | 2015 | 67 |
| KDG | 2016 | 99 |
| KDG | 2017 | 122 |
| KDG | 2018 | 132 |
| KDG | 2019 | 85 |
| KDG | 2020 | 145 |
| TAM | 2019 | 68 |
| MAK | 2019 | 78 |
| TBK | 2019 | 37 |

**Additional Table 2** Rank analysis correlation coefficients for each of the genetic metrics against incidence. The Kendall rank correlation coefficient was obtained by comparing the transmission ranks based on the averages seen at each site. Yule’s *Q* measures the degree of correlation after grouping transmission ranks into two groups (high transmission [transmission rank < 8] and low transmission [transmission rank ≥8]. A Yule’s *Q* of 0 corresponds to no correlation and 1.0 corresponds to maximum correlation. The *p*-value for Yule’s *Q* was assessed using the Pearson chi-squared test.

| **Metric** | **Kendall rank correlation coefficient** | **p-value (Kendall)** | **Yule's Q** | **p-value**  **(Pearsons chi-square)** |
| --- | --- | --- | --- | --- |
| Polygenomic Fraction | 0.3 | 0.12 | 0.8 | 0.14 |
| *The REAL McCOIL COI* | 0.32 | 0.1 | 0.47 | 0.62 |
| *R_H_* | 0.22 | 0.27 | 0.47 | 0.62 |
| Cotransmission Fraction | 0.27 | 0.17 | 0.8 | 0.13 |
| The fraction of non-unique monogenomic clones | 0.38 | 0.04 | 0.96 | 0.01 |
